# Self-Assembled Supramolecular Micelles Based on Multiple Hydrogen Bonding Motifs for the Encapsulation and Release of Fullerene

**DOI:** 10.3390/polym14224923

**Published:** 2022-11-15

**Authors:** Cheng-Wei Huang, Ya-Ying Chang, Chih-Chia Cheng, Meng-Ting Hung, Mohamed Gamal Mohamed

**Affiliations:** 1 Department of Chemical and Materials Engineering, National Kaohsiung University of Science and Technology, Kaohsiung 80778, Taiwan; 2 Department of Applied Chemistry, National Chiao Tung University, Hsinchu 30010, Taiwan; 3 Graduate Institute of Applied Science and Technology, National Taiwan University of Science and Technology, Taipei 10607, Taiwan; 4 Department of Materials and Optoelectronic Science, College of Semiconductor and Advanced Technology Research, Center for Functional Polymers and Supramolecular Materials, National Sun Yat-Sen University, Kaohsiung 804, Taiwan; 5 Chemistry Department, Faculty of Science, Assiut University, Assiut 71515, Egypt

**Keywords:** supramolecular micelle, C_60_, hydroxyl radical scavenger

## Abstract

Living creatures involve several defense mechanisms, such as protecting enzymes to protect organs and cells from the invasion of free radicals. Developing antioxidant molecules and delivery systems to working with enzymes is vital. In this study, a supramolecular polymer PNI-U-DPy was used to encapsulate C_60_, a well-known antioxidant that is hard to dissolve or disperse in the aqueous media. PNI-U-DPy exhibits characteristics similar to PNIPAM but could form micelles even when the environment temperature is lower than its LCST. The U-DPy moieties could utilize their strong complementary hydrogen bonding–interaction to create a physically crosslinked network within PNIPAM micelles, thus adjusting its LCST to a value near the physiological temperature. Morphological studies suggested that C_60_ could be effectively loaded into PNI-U-DPy micelles with a high loading capacity (29.12%), and the resulting complex PNI-C_60_ is stable and remains temperature responsive. A series of measurements under variable temperatures was carried out and showed that a controlled release process proceeded. Furthermore, PNI-C_60_ exhibits hydroxyl radicals scavenging abilities at a low dosage and could even be adjusted by temperature. It can be admitted that the micelle system can be a valuable alternative for radical scavengers and may be delivered to the desired position with good dispersibility and thermo-responsivity. It is beneficial to the search progress of scientists for drug delivery systems for chemotherapeutic treatments and biomedical applications.

## 1. Introduction

Medical technology is constantly improving to combat the harm caused by diseases to human beings. Among these techniques, the drug delivery system is critical in treating various conditions. Scientists have worked hard to develop drug carriers that are simple, accessible to process, have high stability, good biocompatibility, and are sensitive to environmental stimuli. As a proven effective drug carrier, micelles have been reported in quite a few works in the literature [1,2]. Generally, micelles are formed using multi-block copolymers or amphiphilic macromolecules as their main structures. These polymers can self-assemble in aqueous media and include various morphologies, such as core–shell type micelles, rods, cylinders, and vesicles [3,4]. Among these microstructures, micelles are very suitable for entrapping hydrophobic compounds such as drugs since their inner hydrophobic core can isolate them from the external medium [5,6]. It was also reported that when environmental factors (such as temperature, pH, etc.) were changed, the micelles changed their structure so that the drug could be released [7]. However, the synthesis procedure of multi-block copolymers is usually more complicated. The polymeric micelles formed via covalent bonds often exhibit inefficient stability, low stimuli response, and poor drug release rates [8,9], limiting their practical usage in clinical treatments.

On the other hand, polymers with supramolecular interactions, as another way to form micelles, have attracted extensive attention in recent years [10]. This kind of material can be assembled into micelles of different sizes, and the stimuli-responsivity can be manipulated by the strength of interactions [11]. The physical crosslinking network offered by supramolecular functionalities may contribute to a more stable system in the aqueous media compared to traditional materials. The continued dedication to developing supramolecular micelles is of considerable value for drug delivery and chemotherapy research.

Free radicals, a critical species in nature, are involved in many functions of the organism to maintain good health. Reactions involving free radicals can also be observed in other fields, such as atmospheric and polymer chemistry. Even though our bodies produce some antioxidants, excessive free radicals may still affect the biosystem. Reactive oxygen species (ROS), such as hydrogen peroxide and hydroxyl radicals, may cause severe and irreversible damage to tissues and organs [12]. Efficient and safe free radical scavengers were persuaded. Fullerenes, such as C_60_ and C_70_, exhibited high electron affinities and reactive exteriors and are the ideal candidate for quenching ROS [13]. However, the poor water solubility strictly limited the applications of fullerenes in the biomedical field. Chemical modification is one of the common approaches to improving their solubility. Nevertheless, the derivatives and isomers produced after the change may lead to uncertain toxicity. There were some strategies to encapsulate C_60_ to fabricate fullerene-involved composites, such as through carbohydrate polymers [14], silver nanoparticles [15], and metal-organic frameworks (MOFs) [16]. The hybrid materials and encapsulation techniques can be used for drug release, electrode fabrication, and guest molecule adoption [14,15,16]. Another way is toward supramolecular complexation, but this still suffers from low complex stability and a low drug loading content (DLC)(~1 wt%) [13]. Wang and co-workers presented a thermo-responsive polymer P(NIPAM-co-CD), which could form a supramolecular complex by self-assembling β–cyclodextrin and adamantane. This idea could incorporate C_60_ into an aqueous medium [17]. This complex can scavenge hydroxyl radicals, and the antioxidative property can be controlled by temperature. Eom and co-workers recently prepared fullerene-containing nanoparticles through solvophobic and aromatic interactions, followed by a core crosslinking process, which led to a high DLC (~8.12 wt%) of C_60_. However, even if covalent cross-linking provides more stability in the core, it still brings some practical limitations. On the other side, physical cross-linking via non-covalent interactions, such as H–bonding or π–π interactions, are spontaneous and can be accomplished under relatively mild conditions [18]. Therefore, establishing a strategy to improve the water solubility and to reach the target of the controlled release of C_60_, especially through non-covalent interactions, has a significant value in expanding the application of fullerenes.

Polymers with supramolecular interactions have been developed as an ideal strategy to form physically crosslinked micelles. It was proposed that *N*-(6-(3-(2,4-dioxo-3,4-dihydropyrimidin-1(2H)-yl)propanamido)pyridin-2-yl)undec-10-enamide (U-DPy) was a suitable functionality to form supramolecular polymers due to its sextuple complementary hydrogen–bonding interaction. The strong H–bonding interaction can be proved by ^1^H NMR varian temperature experiment and the ^1^H NMR titration experiment, which is stronger than other base pairs such as adenine-thymine (two-point hydrogen bonding system) and diaminopyridine-uracil (three-point hydrogen bonding system) [19]. It was also mentioned in another work that the Polyethylene glycol (PEG) oligomer with the U-DPy end group can form telechelic supramolecular polymers through the U-DPy:U-DPy interaction, suggesting that the recognition units possessing an extremely high *K*_a_ could lead to new supramolecular polymers in bulk as well as in solution [20]. In the previous study, we proposed two supramolecular poly(N-isopropylacrylamide) (PNIPAAm), PNI-DAP and PNI-U-DPy, which contained quadruple and sextuple hydrogen bonding groups on the polymer side chain, respectively [9]. The association constant (*K*_a_) of the DAP:DAP complex was 170 M^−1^ [21], and U-DPy:U-DPy was over 10^6^ M^−1^ [19]. The significant difference of *K*_a_ between the two hydrogen bonding pairs led to totally different properties of two PNIPAAm derivatives, such as the value of a lower critical solution temperature (LCST), particle sizes (PNI-DAP: 38 nm; PNI-U-DPy: 164 nm), kinetic stabilities under surfactants, and micelle morphologies [9]. The strong hydrogen–bonding interaction can also be evidenced by the increase in the polymer’s *T*_g_, the supramolecular aggregation in the GPC trace, the decrease in LCST, and the increased stability against long-time SDS treatment. Owing to the strong self-complementary hydrogen–bonding interaction, PNI-U-DPy exhibits excellent micelle stability and drug loading capacities, which are suitable for application as a drug carrier. We have studied PNI-U-DPy as a carrier to load planar compounds such as doxorubicin (DOX). It was proved that the loaded micelle could successfully endocytose cancer cells [9]. However, to our knowledge, there are few examples of using supramolecular polymeric micelles to encapsulate 3D molecules such as fullerene. Herein, we proposed an efficient and rapid route to use PNI-U-DPy to load C_60_, which is difficult to load and hard to use without chemical modification. The self-assembly of hydrogen bonding moieties and phase transition behaviors of PNI-U-DPy lead to stable micellar structures in aqueous solutions. These micelles show good C_60_ encapsulation capabilities and can effectively release C_60_ upon heating. The loaded micelles (PNI-C_60_) exhibit hydroxyl radicals and scavenging activities at a low dosage and could even be enhanced by ramping the temperature. Such results suggest the micelle system may have the potential to use as a vehicle for C_60_ radical scavenger and provide the possibility of fullerene dispersion and delivery, which expand its application on biomedical applications.

## 2. Materials and Methods

### 2.1. Materials

The supramolecular polymer PNI-U-DPy was synthesized and characterized in the previous literature [9]. C_60_ (purity 99.5%) was purchased from Alfa Aesar (Ward Hill, MA, USA). Organic solvents were purchased from TEDIA (Fairfield, OH, USA) and used as received. DMF was distilled over CaH_2,_ and THF was refluxed with sodium lumps and distilled to remove water. All other reagents not mentioned were obtained from Sigma–Aldrich (St. Louis, MO, USA), Alfa Aesar (Ward Hill, MA, USA), Showa (Osaka, Japan), or TCI (Tokyo, Japan) without further purifications.

### 2.2. Characterization

The molecular weight information, such as the number of the average molecular weight (M_n_), weight average molecular weight (M_w_), and polydispersity (PDI) was investigated by Waters 510 gel permeation chromatography (GPC) system equipped with three Ultrastyragel^TM^ columns (100, 500, and 1000 Å), and Dimethylformamide (DMF) was used as the eluent at a flow rate of 1.0 mL min^–1^ at 50 °C. Polystyrene standards with a narrow PDI were used to calibrate the GPC system. UV-Vis spectra were recorded by HP 8453 spectrometer in the concentration described in the following paragraph. The measurements proceeded with a scanning speed of 200 nm/min and UV-Vis bandwidth of 1.5 nm. The recording wavelength ranged from 190 nm to 1100 nm with a sampling interval of 0.9 nm. Each sample was measured three times to make sure the result was consistent. Dynamic light scattering (DLS) measurements were performed by Brookhaven Instrument Corporation 90-plus at a scattering angle of 90°. C_60_-loaded micelle samples for DLS measurement were prepared in 1,1,2,2-tetrachloroethane (TCE) and first dialyzed against *N,N*-Dimethylacetamide (DMAC), then dialyzed against DI water. Transmission electron microscopy (TEM) images were recorded using an FEI Tecnai T12 microscope operating at 120 kV, and the Image J program was utilized to calculate the number and diameter of micelles from TEM micrographs. Samples for the TEM observation were prepared using micelle dispersant in DI water, which was dropped onto the carbon-coated copper grids and dried under 10 °C for 72 h. The sample of the PNI-U-DPy micelle was stained with RuO4, and the C_60_-loaded micelle samples were observed without stain. The micelle morphologies were observed with Hitachi S-4200 field-emission scanning electron microscopy (FE-SEM, Hitachi, Tokyo, Japan), and the samples were sputtered with Pt before imaging. Wide-angle-X-ray scattering (WAXS) patterns were performed using the wiggler beamline BL17A1 of the National Synchrotron Radiation Research Center (NSRRC), Taiwan, and the radiation with a wavelength of 1.33 Å was carried out under room temperature. Atomic force microscopy (AFM) images were recorded in tapping mode with Dimension 3100 (Digital Instrument) equipped with silicon cantilevers (PPP-NCH-50, 204–497 kHz, 10–130 N/m) at room temperature. The scan rate was 0.5 Hz with a tip velocity of 5 μm/s. The resonance frequency was 394.8 kHz, and the spring constant was 42 N/m. All images were subjected to a first-order plane-fitting procedure to compensate for the sample tilt.

### 2.3. Preparation of C_60_-Loaded Micelles

To prepare C_60_-loaded micelles, PNI-U-DPy were first dissolved, and C_60_ was suspended in TCE with a concentration of 6 mg mL^−1^, respectively, then mixed in a 5:1 ratio and stirred for 10 min. The solution was transferred to an MWCO 6–8 kDa dialysis bag and dialyzed against *N*,*N*-Dimethylacetamide (DMAC) for 48 h, then dialyzed against DI water for another 48 h to remove the organic solvent. The resulting dispersion was centrifuged at 1000 rpm for 10 min to give a transparent supernatant. The supernatant was lyophilized for 24 h, then redispersed and diluted with DI water or a pH 7.4 Phosphate buffered saline (PBS) solution.

### 2.4. Determination of Fullerene Loading

The lyophilized product was dissolved in TCE with sonication. After 10 min, the absorbance of the mixture was recorded by the UV-Vis spectrometer at 410 nm and compared to the calibration curve for C_60_ in TCE (Figure S2 and Table S1).

The drug loading (DL) and encapsulation efficiency (EE) of the micelles were determined using Equations (1) and (2):

(1) $$\mathrm{DL}=\frac{mass\;of\;drug\;encapsulated\;in\;micelles}{mass\;of\;drug-loaded\;micelles}\times 100\%$$

(2) $$\mathrm{EE}=\frac{mass\;of\;drug\;encapsulated\;in\;micelles}{the\;initial\;mass\;of\;drug\;before\;dialysis}\times 100\%$$

### 2.5. Hydroxyl Radical (^●^OH) Scavenging Activity

To determine the interception of hydroxyl radicals by the C_60_-loaded micelles, we measured the UV absorption of Rhodamine-B (Rh-B) in the Fenton reaction. Based on Equation (3), hydrogen peroxide could dissociate into hydroxide ions (OH^−^) and hydroxyl radicals (^●^OH) at low pH in the presence of ferrous ions (Fe^2+^) [22,23].

(3) Fe2++H2O2 →Fe3++OH−+OH●

First, we prepared the Rh-B solution with different concentrations, then mixed it with 25 mM FeSO_4_ solution with the ratio of 2.8:0.2 (*v/v*). The calibration curve was set up by measuring the UV absorption at 554 nm with different concentrations. The radical scavenging experiment was carried out by mixing 0.012 mM Rh-B solution (2.8 mL), PNC_60_, 25 mM FeSO_4_ (0.1 mL), and 6.25 mM H_2_O_2_ (0.1 mL) together. The UV absorption was measured and compared to the calibration curve described above to calculate the scavenging activity. The molar concentration of PNC_60_ is defined by C_60_. The percentage inhibition was calculated with Equation (4) using the following expression [24]:

(4) $$\mathrm{Inhibition}\%\;=\frac{\left|A_{\mathrm{control}}-A_{\mathrm{sample}}\right|}{A_{\mathrm{control}}}\times 100\%$$

where A_control_ = absorbance of the control (containing all reagents except the test compound) and A_sample_ = absorbance of samples (containing all reagents including the test compound). Hydroxyl radical (^●^OH) scavenging activity under different temperatures was measured with a similar procedure. However, the mixture’s temperature was kept at a specific value (26, 28, 30, 32, 34, 36, 38, 40, 44 °C) for 5 min before the H_2_O_2_ solution was added. The scavenging activity was calculated with the method mentioned above.

## 3. Results and Discussion

### 3.1. Preparation and Characterization of PNI-U-DPy and Supramolecular Micelles

**Scheme 1:**
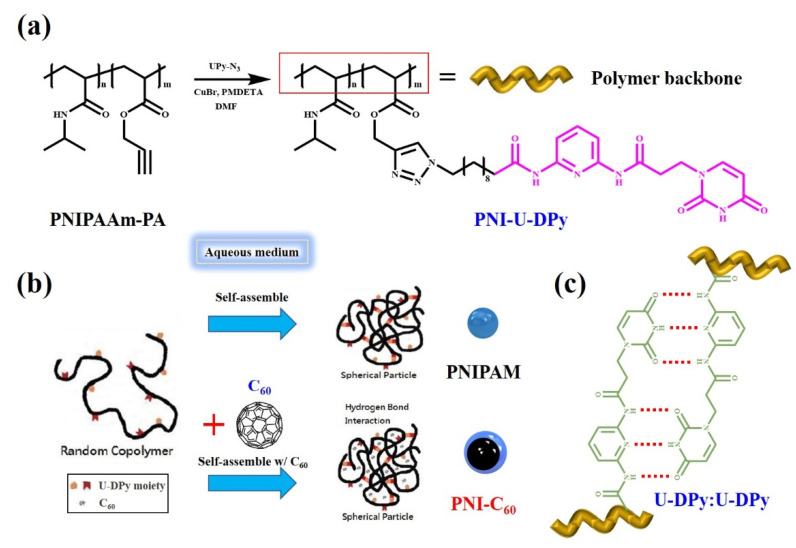
(**a**) Synthesis of PNI-U-DPy; (**b**) Schematic illustration of supramolecular micelles formed by the strong U-DPy:U-DPy complementary multiple hydrogen bonding–interactions and the formation of C_60_-loaded micelles; (**c**) Illustration of complementary hydrogen–bonding interaction within PNI-U-DPy.

**Figure 1:**
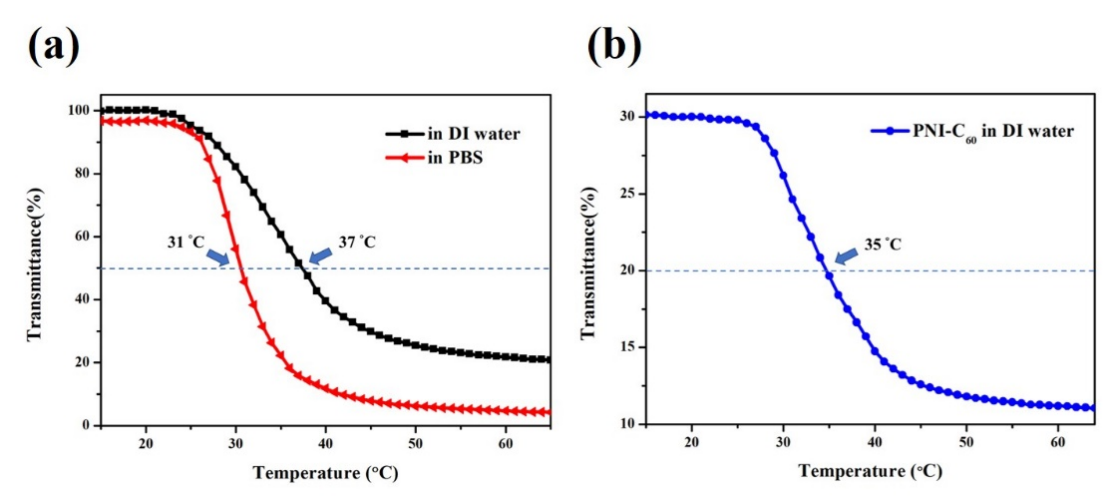
Temperature-dependent transmittance curves at 600 nm of (**a**) PNI-U-DPy in DI water and PBS; (**b**) C_60_-loaded PNI-U-DPy micelles (PNI-C_60_). The blue dotted line represented the 50% transmittance value in the UV-Vis spectra.

The preparation of the supramolecular polymer PNI-U-DPy and micelles were briefly depicted in Scheme 1. A precursor polymer with the propargyl group (PNIPAAm-PA) was “clicked” with UPy-N_3_, a compound containing the azide group and U-DPy hydrogen bonding motif, to give a PNIPAAm derivative with a complementary sextuple hydrogen bonding group at the side chain [9]. The PDI of PNI-U-DPy is 5.97, and *M*_n_ is 18,073. The relatively broad PDI is caused by the multimodal distribution observed at the GPC measurement, which was attributed to the formation of aggregation by the strong hydrogen–bonding interaction and has been described before [9]. The pendant group containing the U-DPy moiety only occupied 5.1% of the whole amount of the repeat unit. This is different from PNIPAM, which can only form an extended coil in water when the temperature is lower than its LCST. PNI-U-DPy was proved to form stable micelles with a low critical micelle concentration (CMC) in the aqueous media due to its strong hydrogen–bonding interaction (Scheme 1c). The LCST of PNI-U-DPy was investigated by observing the turbidity under different temperatures in the DI water and PBS solution, respectively (Figure S1). After calculating the 50% transmittance value from each curve (Figure 1a), the obtained LCST of PNI-U-DPy was 37 °C in DI water and 31 °C in the PBS buffer, which is lower than PNIPAAm’s LCST due to the strong supramolecular network contributed by U-DPy moities [9]. During the temperature ramping process, the forming PNI-U-DPy micelles still exhibited a phase transition ability at temperatures higher than LCST. The transition of PNI-U-DPy in PBS was sharper and faster than in DI water, which may explain the different hydration behaviors between water molecules and PNI-U-DPy in the two media [25]. It also suggested that PNI-U-DPy can form a more stable physically crosslinked network in PBS.

To investigate the thermal responsivity after drug encapsulation, we used the same method to monitor the release profile of C_60_-loaded PNI-U-DPy micelles (PNI-C_60_) (Figure 1b). The lyophilized PNI-C_60_ was redispersed in DI water with 5 mg mL^−1^. There was no precipitate during the redisperse process, suggesting that the structure of the PNI-U-DPy micelle was strong enough to sustain the C_60_ inside the micelles. As shown in Figure 1b, the change in the transmittance profile of PNI-C_60_ is similar to the PNI-U-DPy obtained in the DI water. However, it should be mentioned that after loading, the transmittance of PNI-C_60_ becomes relatively low (~30%) at 600 nm because C_60_ could absorb the radiation at visible light [26,27]. PNI-C_60_ releases half of C_60_ at 35 °C, near the original LCST of PNI-U-DPy, suggesting its suitability and non-selectivity as a drug loading and release system.

### 3.2. C_60_ Encapsulation

**Figure 2:**
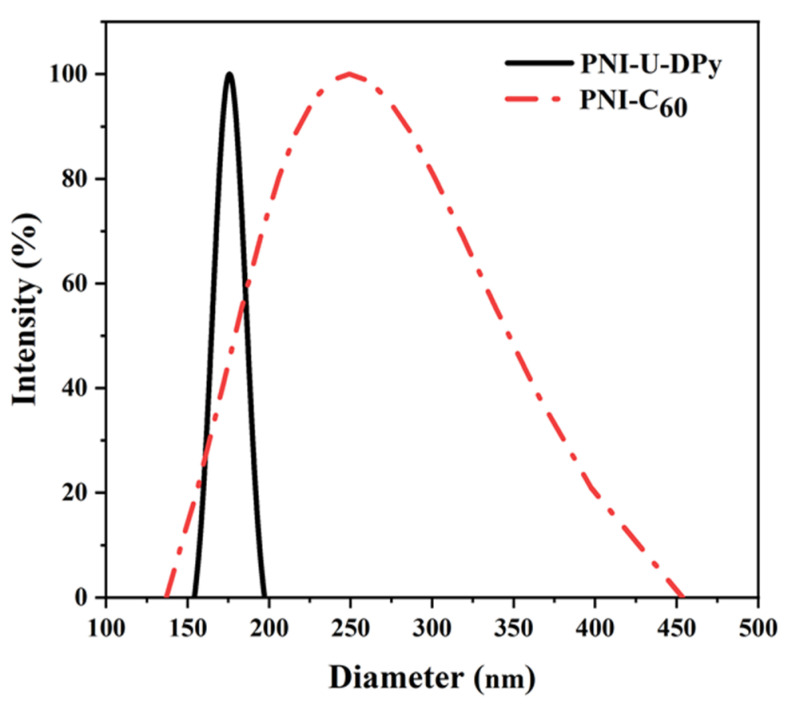
DLS analyses results of PNI-U-DPy micelles and PNI-C_60_ micelles.

PNI-U-DPy can form stable micelles due to the dimmerizable side-chain U-DPy functionality. It also exhibits suitable LCST near the physiological temperature, which is especially important for drug delivery systems. Here, we proceeded with the C_60_ encapsulation experiment by mixing the C_60_ solution with PNI-U-DPy solution, then removing the organic solvent and transferring the mixture to DI water through dialysis, as mentioned above. Because C_60_ has an intrinsic hydrophobic nature, which may aggregate into the PNI-U-DPy micelles’ hydrophobic core, we successfully encapsulated C_60_ during the PNI-U-DPy self-assembly process in water. The comparison of the size of the PNI-U-DPy micelles before and after C_60_ loading is shown in Figure 2. When C_60_ was loaded into PNI-U-DPy, the resulting micelle composite PNI-C_60_ exhibited a broad single distribution with a PDI of 0.177 ± 0.013, which was significantly different from the result obtained from the PNI-U-DPy micelles. Additionally, the particle size increased from 170 nm to 345 nm after loading, which suggests that C_60_ was successfully encapsulated into the micelles. To calculate the C_60_-loading efficiency, we first set up the calibration curve by dissolving C_60_ in TCE under different concentrations and measuring the absorbances with a UV-Vis spectrometer (Figure S2). The lyophilized PNI-C_60_ was then dissolved in TCE and measured for UV-Vis absorbance, followed by calculations with Equations (1) and (2). The DLC is 29.12%, and EE is 5.83%, comparable with the proposed PNIPAM micelles [28] loading with the drug in a planar structure such as doxorubicin (DOX). The moderate loading efficiency compared to other carrier systems, such as MOFs, may be due to the nature of the smaller size and surface area of the micelle carrier [29]. However, it should be mentioned there is relatively little in the literature on unmodified C_60_ encapsulating with micelles. It is difficult to find comparison standards from other studies. Thus, the excellent DLC and EE value for micelles may lead to easier control of the loading content actually needed.

**Figure 3:**
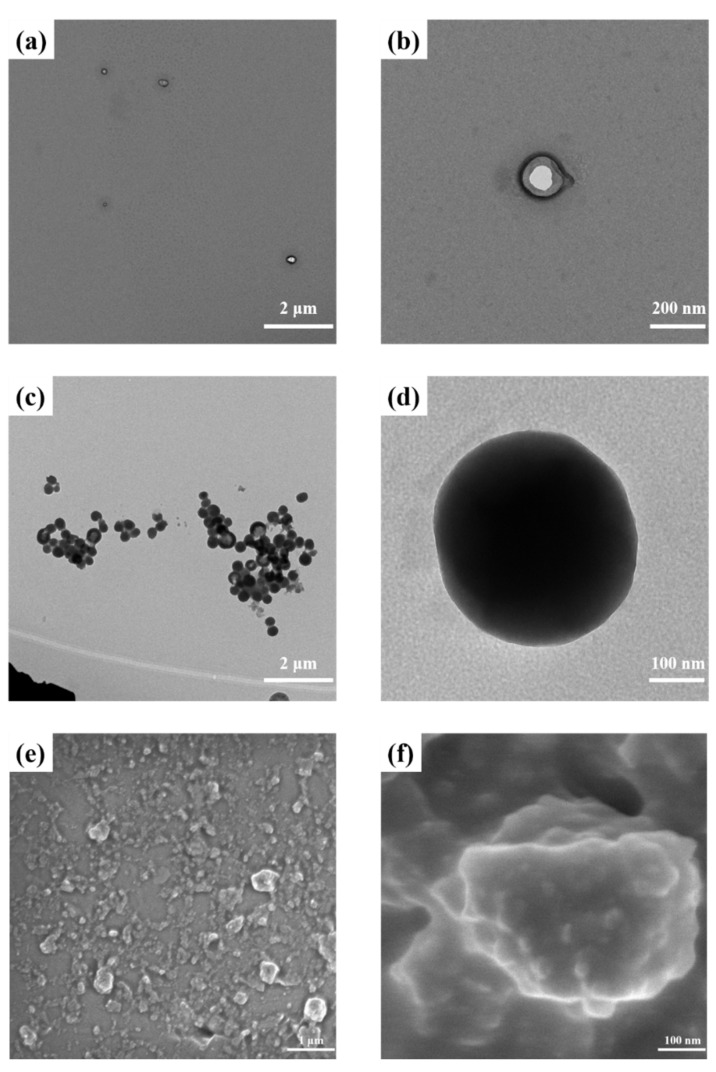
TEM and SEM images of the micelles: (**a**) TEM of PNI-U-DPy micelles; (**b**) TEM of PNI-U-DPy micelles (magnified); (**c**) TEM of PNI-C_60_ micelles; (**d**) TEM of PNI-U-DPy micelles (magnified); (**e**) SEM of PNI-C_60_ micelles; (**f**) SEM of PNI-C_60_ micelles (magnified).

**Figure 4:**
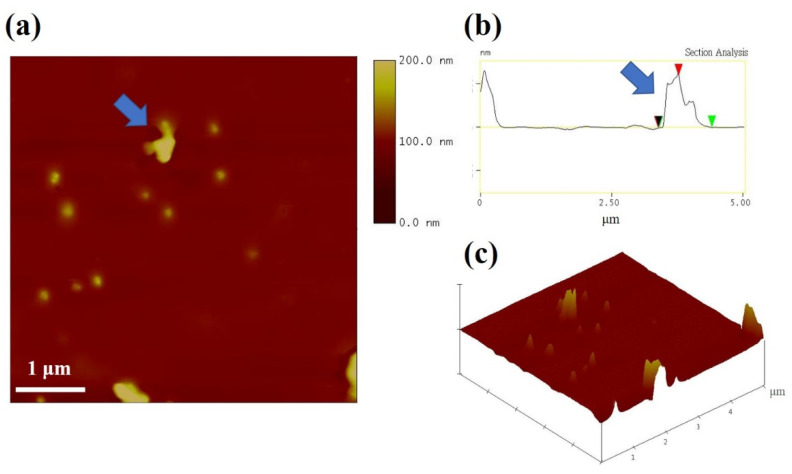
AFM images of the PNI-C_60_: (**a**) Top-view; (**b**) Section analysis; (**c**) Side-view. The blue arrows show a PNI-C_60_ which was used to calculate the diameter. The black, red, and green marks in Figure 4b was used to measure the horizontal distance and vertical distance.

To investigate the morphology of PNI-C_60_, we prepared samples by redispersing the lyophilized PNI-C_60_ in the water and dropping it onto silicon wafers and carbon-coated copper grids for SEM and TEM measurements, respectively. PNI-U-DPy micelles were also used to compare in TEM observation, which was stained with RuO_4_. The corresponding images were collected, as shown in Figure 3. The TEM image of C_60_ is shown in Figure S3. PNI-U-DPy micelles exhibit vesicle-like structures with average diameters near the DLS measurement values (Figure 3a,b). In contrast, PNI-C_60_ micelles show different morphologies from the PNI-U-DPy micelles (Figure 3c,d). The interior of the micelles became extremely dark compared with the PNI-U-DPy micelles, which suggested that the high electron density species, C_60_, were encapsulated. The mean size of the complex calculated by the 76 micelles in the TEM image (Figure 3c) is 342 ± 7 nm, which was in good agreement with the DLS results. Additionally, we can observe that several PNI-C_60_ micelles may gather closely to form large aggregates, but the structure did not collapse to form huge micelles, indicating that the PNI-U-DPy micelles were stable enough to maintain the network even in the dialysis process due to the strong–hydrogen bonding interaction. SEM images of PNI-C_60_ also show the aggregate formed from tiny micelles (Figure 3e,f). AFM images were recorded with a drop-coated sample, as shown in Figure 4. The height of the complex is 79 ± 20 nm, which was calculated by the AFM z-scale measure results. The mean size calculated from the AFM image is 212 ± 125 nm. It presents similar results to SEM, which contains particles with a diameter ranging from 150 nm to 350 nm. The combined results of DLS, TEM, SEM, and AFM prove the formation of the supramolecular micelle complex.

**Figure 5:**
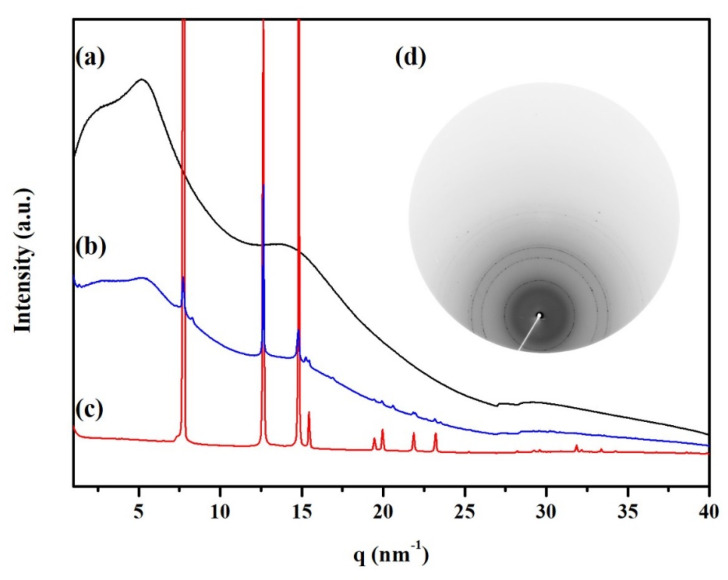
WAXS profiles of (**a**) PNIPAM-PA; (**b**) PNI-C_60_; (**c**) C_60_ and (**d**) Two-dimensional WAXS image of PNI-C_60_.

C_60_ was reported to show strong crystallinity even in the solvated state [30,31,32]. Therefore, we decided to use the wide-angle X-ray technique to check the crystalline behavior of the samples discussed in this study. As shown in Figure 5 and Figure S4, PNIPAM-PA displays a classic amorphous pattern without any crystalline peaks. In contrast, C_60_ reveals a series of crystalline peaks that contributes to its FCC crystalline nature [32]. Interestingly, PNI-C_60_ exhibits a mixed state containing both amorphous and crystalline peaks. The broadband should be offered by the PNI-U-DPy, which is an amorphous polymer, while the intense peak is from C_60_. The 2D WAXS images also imply crystalline in the interior of the PNI-C_60_ micelles (Figure 5d and Figure S4). When the C_60_ was encapsulated by the micelles, it still maintained a partial crystallinity since there were several peaks in the WAXS pattern. However, it contains not only FCC but other crystalline structures, as shown in Figure 5b. It may be explained by the close aggregation of C_60_ inside the PNI-U-DPy micelles, which was indicated through the TEM observation results. Accordingly, the encapsulation of C_60_ through the PNI-U-DPy micelles remained crystalline, which may offer a new strategy for fabricating the bottom-up fullerene materials [33].

### 3.3. Drug Release Study of Micelles

**Figure 6:**
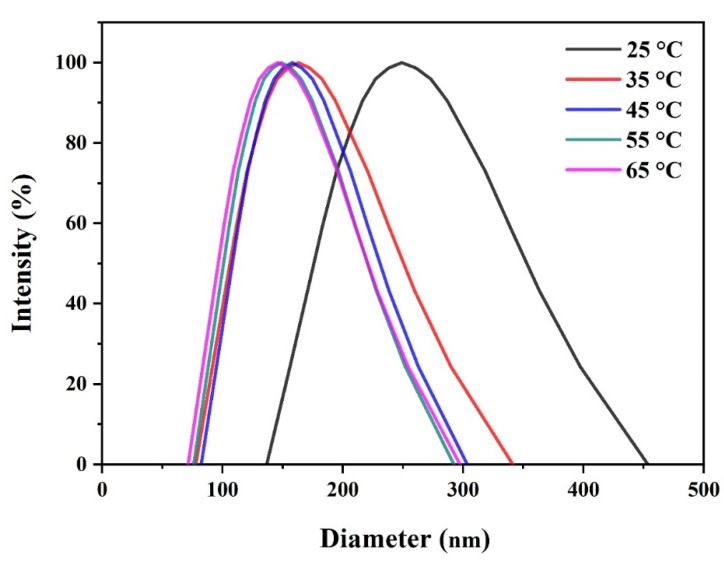
DLS measurement of PNI-C_60_ micelles under different temperatures.

**Figure 7:**
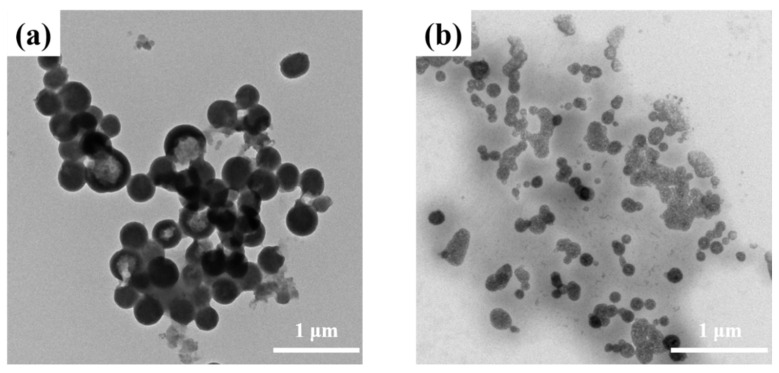
TEM images of (**a**) PNI-C_60_ micelles and (**b**) PNI-C_60_ micelles after heating.

A fast and controlled release behavior is quite essential for a drug delivery system. We have confirmed that PNI-U-DPy exhibits LCST near the physiological temperature above. Here, we continued to discuss the release behavior of PNI-C_60_ through morphological analyses. The variable-temperature of the DLS measure result was carried out from 25 °C to 65 °C (Figure 6). As described before, PNI-C_60_ exhibits a diameter of 345 nm at room temperature, with a relatively broad polydispersity (Table 1). The diameter dramatically changed to 268 nm when the temperature increased to 35 °C, near the LCST of PNI-U-DPy. While the temperature became higher, the diameter continued to decrease, though only slightly. The diameter seems to be gradually tending to a value of about 220~230 nm when the temperature is over 45 °C. This indicates that most encapsulated C_60_ may be released between the temperature range of 35 °C to 45 °C. On the other hand, TEM observation of the samples prepared under 25 °C and heated at 45 °C is collected in Figure 7. The sample prepared with an anneal process shows a collapsed structure that did not contain a dark interior as the sample prepared at 25 °C. Furthermore, the size of the micelles decreased significantly, and some shadow surrounded the released micelles. It further proves that the loading and controlled release of PNI-U-DPy micelle system is successfully proceeding.

### 3.4. Hydroxyl Radical Scavenging Ability

**Figure 8:**
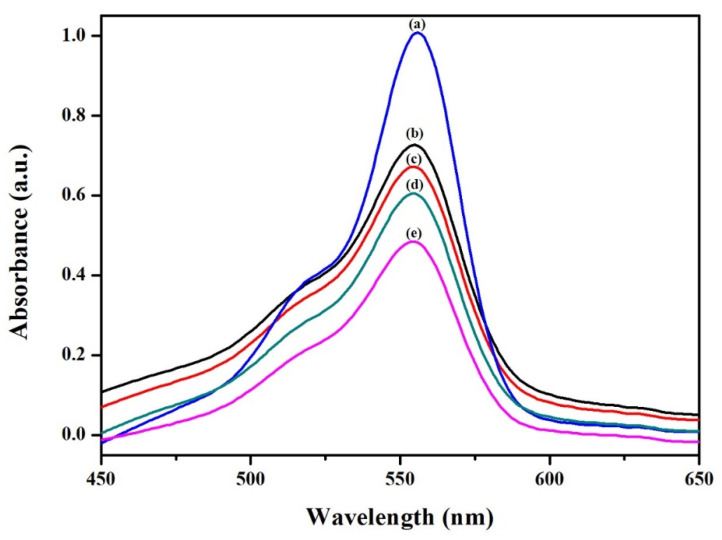
UV-Vis spectra of mixture with different conditions: (**a**) Control sample 1, 0.012 mM Rh-B + 25 mM FeSO_4_; (**b**) 0.012 mM Rh-B + 0.15 mM PNI-C_60_ + 25 mM FeSO_4_ + 500 mM H_2_O_2_; (**c**) 0.012 mM Rh-B + 0.10 mM PNI-C_60_ + 25 mM FeSO_4_; (**d**) 0.012 mM Rh-B + 0.05 mM PNI-C_60_ + 25 mM FeSO_4_ + 500 mM H_2_O_2_; (**e**) Control sample 2, 0.012 mM Rh-B + 25 mM FeSO_4_ + 500 mM H_2_O_2_.

**Figure 9:**
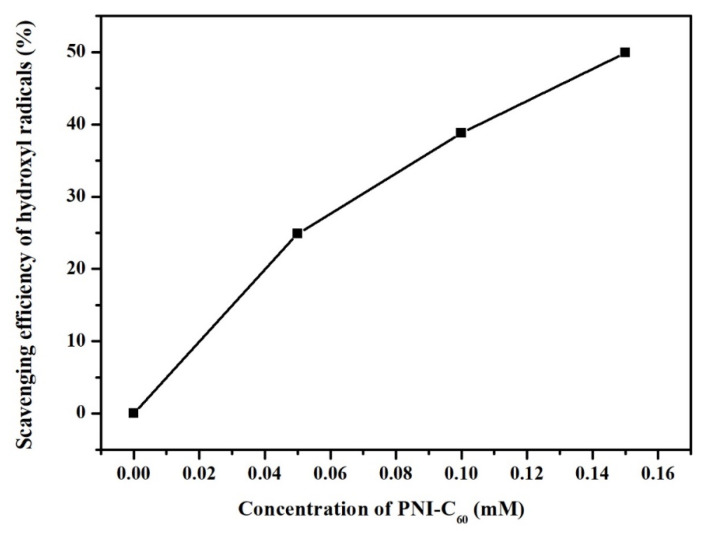
Hydroxyl radical scavenging efficiency of PNI-C_60_ in Fenton reaction at different concentrations.

The hydroxyl radical scavenging activity of PNI-C_60_ was evaluated through the Fenton reaction [34]. According to the literature, because fullerene species could quench the fluorescence of Rh-B, the analyses were recorded through a UV-Vis spectrometer [17]. Rh-B shows a strong absorption at 554 nm in the aqueous medium [35], and the absorption will decrease when the structure of Rh-B has been reacted by the hydroxyl radicals. PNI-C_60_ was used as an antioxidant to protect Rh-B against oxidation. A PBS solution with 0.1 M EDTA was used as a solvent in the experiment. Stock solutions of 0.012 mM Rh-B, 25 mM FeSO_4,_ and 500 mM H_2_O_2_ were prepared. The Rh-B solution, PNI-C_60_ dispersion, FeSO_4_ solution, and H_2_O_2_ solution were added step by step in sequence into a quartz cell and were stirred for 10 s, followed by UV-Vis measurement. The absorption of Rh-B is very strong for control sample 1(without H_2_O_2_ and PNI-C_60_) since the hydroxyl radical did not generate (Figure 8a). When the H_2_O_2_ was added to the cell (control sample 2), the absorption decreased seriously within 10 s, suggesting that when the hydroxyl radicals were produced and oxidized, the Rh-B molecules immediately decreased (Figure 8e). However, if PNI-C_60_ is present in the mixture, the absorption at 554 nm could be higher than the control sample 2, indicating that PNI-C_60_ may inhibit the oxidation of hydroxyl radicals. Furthermore, we also found that with the increase in the PNI-C_60_ addition amount, the inhibition effect became more evident (Figure 8b–d). The hydroxyl radical scavenging efficiency of different PNI-C_60_ was calculated, as shown in Figure 9. Surprisingly, the scavenging efficiency achieved 40% when only 0.1 mM PNI-C_60_ was added to the assay. The addition is much lower than the previous study [17], signifying that PNI-C_60_ could be used as an effective inhibitor against hydroxyl radicals.

**Figure 10:**
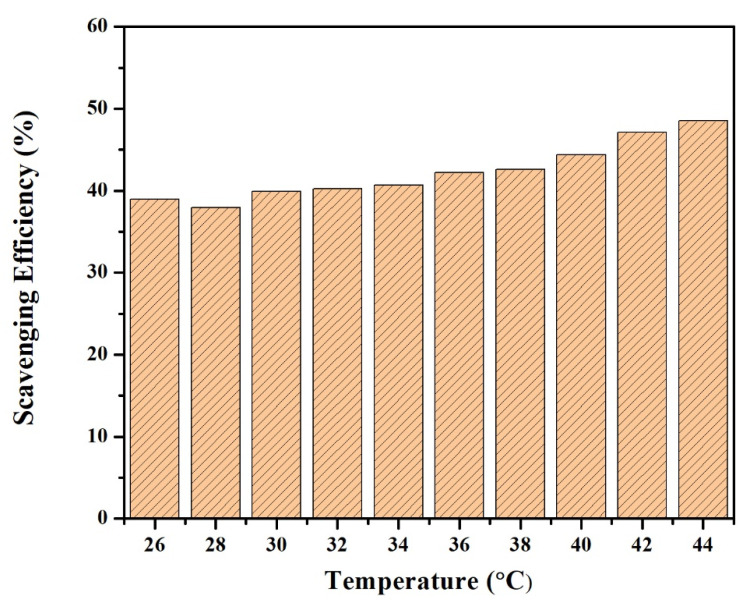
Hydroxyl radical scavenging efficiency of PNI-C_60_ in Fenton reaction from 26 to 44 °C with 0.10 mM PNI-C_60_.

As described in the above paragraph, PNIPAM is a thermo-responsive material, and its LCST could be adjusted by introducing U-DPy functionalities into the polymer side chain. Additionally, the C60-loaded micelles exhibit thermo-responsivity since the size of the micelles decreases after heating, which was proved by DLS and electronic microscopy observations. We continued to explore how the temperature change affected the scavenging property. The experiment was carried out at a PNI-C_60_ concentration of 0.1 mM, and the temperature was changed from 26 to 44 °C. The result was collected and calculated in Figure 10. While the environment temperature is lower than PNI-U-DPy’s LCST, it maintains a scavenging efficiency of around 40%. In contrast, when the temperature exceeds the LCST, the scavenging efficiency starts to increase. This may be explained by the change in morphologies upon heating. As discussed, PNI-C_60_ undergoes the phase transition process, rapidly releasing the encapsulated C_60_ into the mixture and then triggering the fullerene’s inhibition mechanism. Therefore, the results mentioned above give a conclusion that PNI-C_60_ has the potential to act as an antioxidant with thermo-responsivity and a controlled release manner.

## 4. Conclusions

In this study, it was demonstrated that the supramolecular micelles were obtained from a designed polymer PNI-U-DPy. This PNIPAM derivative contains a strong self-complementary hydrogen bonding interaction at the side chain, which constructs a physically crosslinked network and increases the stability of the micelles. The intermolecular interaction leads to an apparent change in LCST and makes it closer to the physiological temperature. It also enables PNI-U-DPy micelles to encapsulate C_60_ with high loading content and entrapment stability, which is seldom reported by unmodified fullerene and polymeric micelle systems. DLS, TEM, SEM, and WAXS observation results suggest that C_60_ has been effectively loaded into a micelle to form a complex hybrid PNI-C_60_. The encapsulated C_60_ exhibits a crystalline behavior inside the supramolecular micelles. The behavior of PNI-C_60_ was investigated under several variable temperature measurements and gave practical proof for the controlled release progress. Due to the existence of C_60_, the PNI-C_60_ complex exhibit hydroxyl radicals and scavenging activity at a low dosage and could even be enhanced by ramping the temperature. These findings show that supramolecular polymers could be a potential candidate as a C_60_ carrier without destroying its structure. This strategy provides the possibility for designing an efficient drug delivery system for those drugs that are difficult to encapsulate. It may also contribute to understanding the chemistry and biological behavior of fullerene in aqueous media, therefore offering a potential route for the fabrication of effective and eco-friendly assays for biomedical applications.

## Figures and Tables

**Table 1:** DLS measure results of PNI-C_60_ under different temperatures.

Temperature	25 °C	35 °C	45 °C	55 °C	65 °C
Dh (nm)	345 ± 3	268 ± 2	247 ± 4	235 ± 2	234 ± 2
polydispersity	0.277 ± 0.01	0.247 ± 0.01	0.227 ± 0.01	0.198 ± 0.01	0.194 ± 0.01

## Data Availability

Data will be available from the first author on demand.

## Supplementary Materials

The following supporting information can be downloaded at: https://www.mdpi.com/article/10.3390/polym14224923/s1, Table S1. The transmittance of C_60_ in TCE; Figure S1. Temperature–dependent transmittance curves of PNI-U-DPy in (**a**) DI water; (**b**) PBS; Figure S2. Calibration curve set up by C_60_ in TCE; Figure S3. TEM image of C_60_; Figure S4. Two-dimensional WAXS image of (**a**) PNIPAM-PA; (**b**) C_60_. [9,19]
