# Supplementary material for: Self-Assembled Supramolecular Micelles Based on Multiple Hydrogen Bonding Motifs for the Encapsulation and Release of Fullerene
Source: Polymers (Basel). 2022 Nov 15;14(22):4923. doi: 10.3390/polym14224923 (PMC9699310; doi:10.3390/polym14224923)
Supplement: Supplementary file 1 [file polymers-14-04923-s001.zip › polymers-2012596-supplementary.pdf]

Supporting Information for

Self-Assembled Supramolecular Micelles Based on Multiple Hydrogen Bonding Motifs for the Encapsulation and Release of C_{60}

Cheng-Wei Huang [1,*], Ya-Ying Chang [2], Chih-Chia Cheng [3], Meng-Ting Hung [1], Mohamed Gamal Mohamed [4,5]

[1] Department of Chemical and Materials Engineering, National Kaohsiung University of Science and Technology, Kaohsiung 80778, Taiwan

[2] Institute of Applied Chemistry, National Chiao Tung University, Hsinchu 30010, Taiwan

[3] Graduate Institute of Applied Science and Technology, National Taiwan University of Science and Technology, Taipei 10607, Taiwan

[4] Department of Materials and Optoelectronic Science, College of Semiconductor and Advanced Technology R search, Center for Functional Polymers and Supramolecular Materials, National Sun Yat-Sen University, Kaohsiung 804, Taiwan

[5] Chemistry Department, Faculty of Science, Assiut University, Assiut 71515, Egypt

* Correspondence: cwhuangs@nkust.edu.tw (C.W.H.).

Synthesis:

The synthetic route to produce PNI-U-DPy is shown in Scheme 1. Detailed steps were described

below.

(1) Polymerization of PNIPAM-PA

Distilled NIPAM (10 g, 88 mmol) was added into a round bottle, following by added CuBr (0.7 mg, 5 mmol), propargyl acrylate (0.7 mL, 6 mmol), PMDETA (0.05 mL, 0.2 mmol), DI water 30 mL and 45 mL DMF. The mixture was then proceeded the freeze-pump-thaw process three times and added Ethyl-2-bromobutyrate (EBB) (0.25 mL, 1 mmol) and kept reacting at 0 °C overnight. The mixture was then passed through an aluminum oxide column and evaporated to remove solvents. The crude product was then precipitated to diethyl ether three times to give a white powder with an 86% yield.

(2) Synthesis of PNI-U-DPy

N_3-U-DPy was obtained by the previously reported procedures[1, 2]. PNIPAM-PA (1 g, 38 μmol) and N_3-U-DPy (10 mg, 2.5 μmol) were first added into a round flask. CuBr (20 mg, 120 μmol), DMF (40 mL) and PMDETA (25 μL, 120 μmol) was added. The mixture then went through a freeze-pump-thaw process, reacted at 60 °C overnight, and dialysis against DMF with a 6000-8000 MWCO dialysis bag for three days and then precipitated in diethyl ether. A white powder was obtained and dried in vacuum to give a yield of 85%.

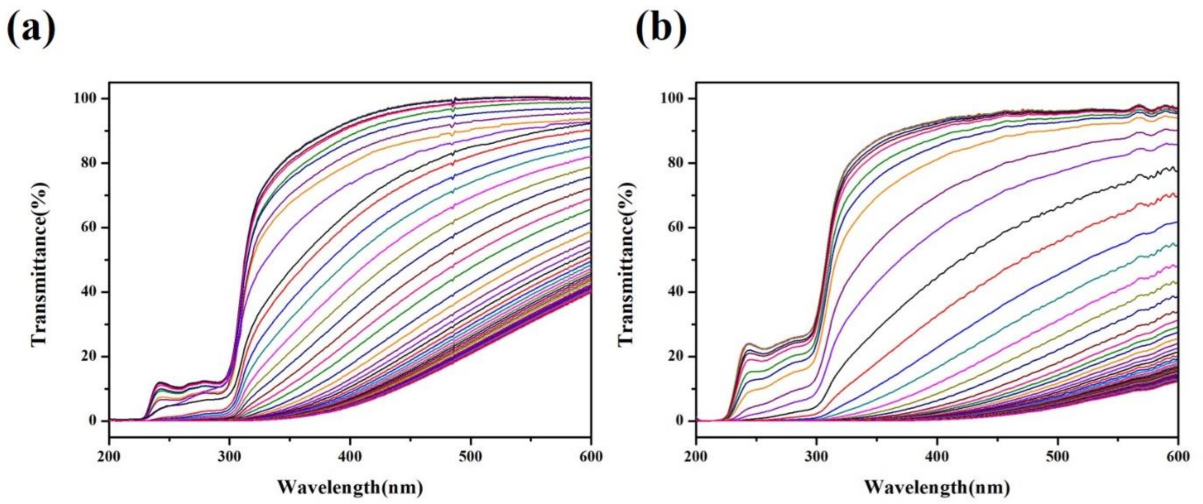

Figure S1. Temperature-dependent transmittance curves of PNI-U-DPy in (a) DI water; (b) PBS.

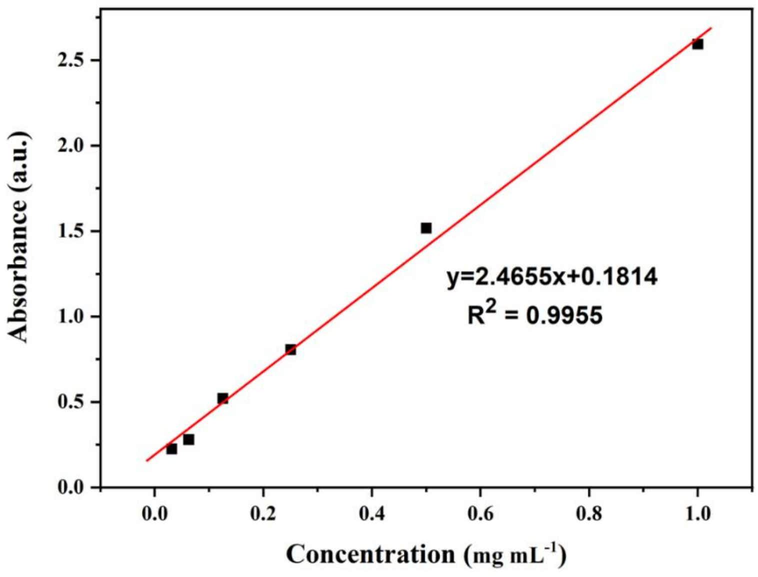

Figure S2. Calibration curve set up by C_{60} in TCE.

Table S1. The transmittance of C_{60} in TCE.

**Concentration (mg/mL)**	**Transmittance at 410 nm**
1	0.3%
0.5	3.0%
0.25	15.6%
0.125	30.2%
0.0625	52.5%
0.03125	59.5%

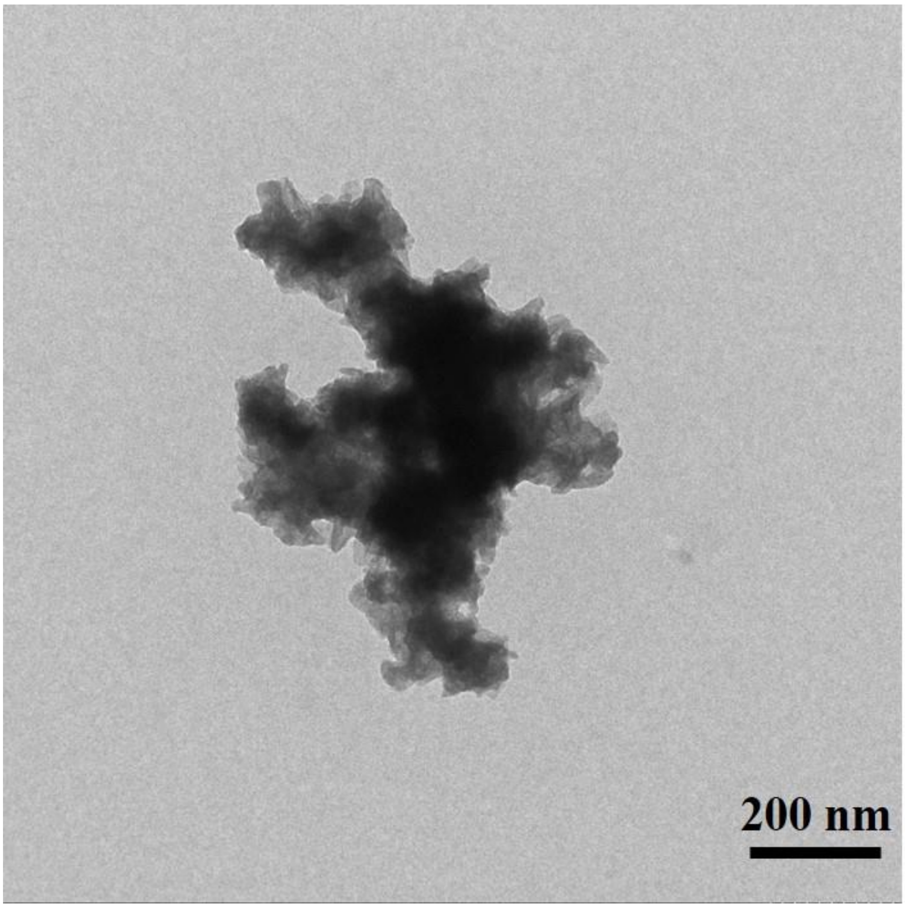

Figure S3. TEM image of C_{60}.

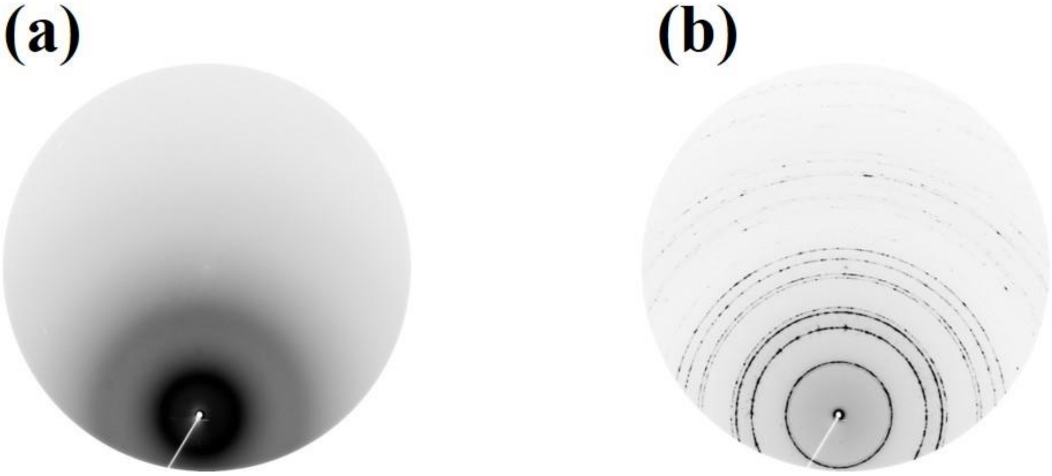

Figure S4. Two-dimensional WAXS image of (a) PNIPAM-PA; (b) C_{60}.

Reference

1. Cheng, C. C.; Chang, F. C.; Kao, W. Y.; Hwang, S. M.; Liao, L. C.; Chang, Y. J.; Liang, M. C.; Chen, J. K. and Lee, D. J., Highly efficient drug delivery systems based on functional

supramolecular polymers: In vitro evaluation. *Acta Biomater*, **2016**, 33, 194-202. https://doi.org/10.1016/j.actbio.2016.01.018.

2. Cheng, C.-C.; Yen, Y.-C. and Chang, F.-C., Hierarchical structures formed from self-complementary sextuple hydrogen-bonding arrays. *RSC Advances*, **2011**, 1, 1190-1194. https://doi.org/10.1039/C1RA00513H.
